# Supplementary material for: Chromothripsis during telomere crisis is independent of NHEJ, and consistent with a replicative origin
Source: Genome Res. 2019 May;29(5):737–49. doi: 10.1101/gr.240705.118 (PMC6499312; doi:10.1101/gr.240705.118)
Supplement: Supplemental Material [file supp_gr.240705.118_Supplemental_file_1.zip › contigs/annotated_contigs/DB80/contig.2.DB80_length_462_mean_cov_9.34848484848.docx]

**DB80_length_462_mean_cov_9.34848484848**

```
TTCTTTTCACCACGCAAATCATCACAAGCTCTTCCTCATTCCAGGAAAACCTTCATAGTGTTCATTCATGATCAGATCAGAAGTAGTGT
 >chr20:40715184-40715363 + E=4e-96
AAAAAATATAGGGGTTTCGGGAGGCGGAGCTTGCAGTGAGCTGAGATCGCGCCACTGCACTCCAGCCTGGGTGACACAGCGAGACTCCA

T|ATATATA|TGTCTCTGTTAAGCCACTAGATAACTTTAGCTGAGGAGAGAATGTTGAGGTGGGGGGTGGGGTGTGTGGCTGCAGAGAG
         >chr20:40716577-40716853 + E=1e-154
GAGCAAGGCATGCTCCCTCCAGTTCCTGGTTCCCAAGGGGCACAAGATGGATCCTGCCTAGGTCTACAGGTCCCATTGCTGGGGTACAG

AGGGGAGACAAGATTGCGGCTGCAGGCTCGCTGGGGACCACCCCAACATGAACAAAGAAGTCATGTGTTAGACCCTTCATCTGGGGGTT

ACCCTGAAGATATAGCCAA
```
